# The GLEaMviz computational tool, a publicly available software to explore realistic epidemic spreading scenarios at the global scale

**DOI:** 10.1186/1471-2334-11-37

**Published:** 2011-02-02

**Authors:** Wouter Van den Broeck, Corrado Gioannini, Bruno Gonçalves, Marco Quaggiotto, Vittoria Colizza, Alessandro Vespignani

**Affiliations:** 1 Computational Epidemiology Laboratory, Institute for Scientific Interchange (ISI), Turin, Italy; 2 Center for Complex Networks and Systems Research, School of Informatics and Computing, Indiana University, Bloomington, IN 47408, USA; 3 Pervasive Technology Institute, Indiana University, Bloomington, IN 47404, USA; 4 Department of Industrial Design, Arts, Communication and Fashion (INDACO), Politecnico di Milano, Milan, Italy; 5 INSERM, U707, Paris F-75012, France; 6 UPMC Université Paris 06, Faculté de Médecine Pierre et Marie Curie, UMR S 707, Paris F75012, France; 7 Institute for Scientific Interchange (ISI), Turin, Italy

## Abstract

**Background:**

Computational models play an increasingly important role in the assessment and control of public health crises, as demonstrated during the 2009 H1N1 influenza pandemic. Much research has been done in recent years in the development of sophisticated data-driven models for realistic computer-based simulations of infectious disease spreading. However, only a few computational tools are presently available for assessing scenarios, predicting epidemic evolutions, and managing health emergencies that can benefit a broad audience of users including policy makers and health institutions.

**Results:**

We present "GLEaMviz", a publicly available software system that simulates the spread of emerging human-to-human infectious diseases across the world. The GLEaMviz tool comprises three components: the client application, the proxy middleware, and the simulation engine. The latter two components constitute the GLEaMviz server. The simulation engine leverages on the Global Epidemic and Mobility (GLEaM) framework, a stochastic computational scheme that integrates worldwide high-resolution demographic and mobility data to simulate disease spread on the global scale. The GLEaMviz design aims at maximizing flexibility in defining the disease compartmental model and configuring the simulation scenario; it allows the user to set a variety of parameters including: compartment-specific features, transition values, and environmental effects. The output is a dynamic map and a corresponding set of charts that quantitatively describe the geo-temporal evolution of the disease. The software is designed as a client-server system. The multi-platform client, which can be installed on the user's local machine, is used to set up simulations that will be executed on the server, thus avoiding specific requirements for large computational capabilities on the user side.

**Conclusions:**

The user-friendly graphical interface of the GLEaMviz tool, along with its high level of detail and the realism of its embedded modeling approach, opens up the platform to simulate realistic epidemic scenarios. These features make the GLEaMviz computational tool a convenient teaching/training tool as well as a first step toward the development of a computational tool aimed at facilitating the use and exploitation of computational models for the policy making and scenario analysis of infectious disease outbreaks.

## Background

The 2009 H1N1 influenza pandemic highlighted the importance of computational epidemic models for the real-time analysis of the health emergency related to the global spreading of new emerging infectious diseases [1-3]. Realistic computational models are highly complex and sophisticated, integrating substantial amounts of data that characterize the population and geographical context in order to attain superior accuracy, resolution, and predictive power [4-10]. The challenge consists in developing models that are able to capture the complexity of the real world at various levels by taking advantage of current information technology to provide an *in silico *framework for testing control scenarios that can anticipate the unfolding of an epidemic. At the same time, these computational approaches should be translated into tools accessible by a broader set of users who are the main actors in the decision-making process of health policy, especially during an emergency like an influenza pandemic. The tradeoff between realistic and accurate descriptions of large-scale dynamics, flexibility, computational feasibility, ease of use, and accessibility of these tools creates a major challenge from both the theoretical and the computational points of view [4,5,11,10,13]. GLEaMviz is a client-server software system that can model the world-wide spread of epidemics for human transmissible diseases like influenza-like illnesses (ILI), offering extensive flexibility in the design of the compartmental model and scenario setup, including computationally-optimized numerical simulations based on high-resolution global demographic and mobility data. GLEaMviz makes use of a stochastic and discrete computational scheme to model epidemic spread called "GLEaM" - GLobal Epidemic and Mobility model, presented in previously published work [6,3,14] - which is based on a geo-referenced metapopulation approach that considers 3,362 subpopulations in 220 countries of the world, as well as air travel flow connections and short-range commuting data. The software includes a client application with a graphical user interface (GUI) for setting up and executing simulations, and retrieving and visualizing the results; the client application is publicly downloadable. The server application can be requested by public institutions and research centers; conditions of use and possible restrictions will be evaluated specifically.

The tool is currently not suitable for the simulation of vector-borne diseases, infection transmission depending on local contact patterns such as sexually transmitted diseases and diseases with a time scale that would make demographic effects relevant. The tool, however, allows the introduction of mitigation policies at the global level. Localized intervention in space or time can be implemented in the GLEaM model and their introduction in the GLEaMviz computational tool are planned for future releases.

Only a few computational tools are currently available to the public for the analysis and modeling of epidemics. These range from very simple spreadsheet-based models aimed at providing quick estimates for the number of patients and hospitalizations during a pandemic (see e.g. *FluSurge *[15]) to more complicated tools based on increasingly sophisticated simulation approaches [11,16,10,13,5]. These tools differ in their underlying modeling approaches and in the implementation, flexibility, and accessibility of the software itself.

*InfluSim *is a tool that provides a visual interface to simulate an epidemic with a deterministic compartmental model in a single population [11]. The model includes age structure and explicit sojourn times with different stages in each compartment, extending an SEIR compartmentalization to include hospitalizations and intervention measures. The software provides the infectious disease dynamics and the user can set parameter values and add or remove interventions. However, no spatial structure or other forms of heterogeneity and stochasticity are considered in the model.

On the other hand agent-based models describe the stochastic propagation of a disease at the individual level, thus taking into account the explicit social and spatial structure of the population under consideration. *CommunityFlu *is a software tool that simulates the spread of influenza in a structured population of approximately 1,000 households with 2,500 persons [13]. User interaction with the software is limited to the spreadsheet portion of the program, where one can choose the type of intervention and other parameters describing the disease and the population.

A larger population is considered in *FluTe*, a publicly available tool for the stochastic simulation of an epidemic in the United States at the level of individuals [10]. The model is based on a synthetic population, structured in a hierarchy of mixing social groups including households, household clusters, neighborhoods, and nation-wide communities. *FluTe *comes with a configuration file in text format that can be modified by an expert user to set various parameters such as the initiation of the epidemic, the reproductive number, and the interventions considered. No GUI is provided, and the output of the simulations is given in the form of text files that must be analyzed through additional software.

*EpiFast *involves a parallel algorithm implemented using a master-slave approach which allows for scalability on distributed memory systems, from the generation of synthetic population aggregated in mixing groups to the explicit representation of the contact patterns between individuals as they evolve in time [5]. The *EpiFast *tool allows for the detailed representation and simulation of the disease on social contact networks among individuals that dynamically evolve in time and adapt to actions taken by individuals and public health interventions. The algorithm is coupled with a web-based GUI and the middleware system Didactic, which allows users to specify the simulation setup, execute the simulation, and visualize the results via plots. Epidemic models and interventions are pre-configured, and the system can scale up to simulate a population of a large metropolitan area on the order of tens of millions of inhabitants.

Another class of models focuses on the global scale, by using a metapopulation approach in which the population is spatially structured into patches or subpopulations (e.g. cities) where individuals mix. These patches are connected by mobility patterns of individuals. In this vein two tools are currently available. The Global Epidemic Model (GEM) uses a metapopulation approach based on an airline network comprised of 155 major metropolitan areas in the world for the stochastic simulation of an influenza-like illness [16]. The tool consists of a Java applet in which the user can simulate a hypothetical H1N1 outbreak and test pre-configured intervention strategies. The compartmentalization is set to an SEIR model, and the parameterization can be modified in the full or stand-alone mode, but not currently in the Java applet.

The Spatiotemporal Epidemiological Modeler (STEM) is a modeling system for the simulation of the spread of an infectious disease in a spatially structured population [16]. Contrary to other approaches, STEM is based on an extensible software platform, which promotes the contribution of data and algorithms by users. The resulting framework therefore merges datasets and approaches and its detail and realism depend on continuous developments and contributions. However, these are obtained from a variety of sources and are provided in different formats and standards, thus resulting in possible problems related to the integration and merging of datasets. Such issues are left to the user to resolve.

The existing tools described above thus offer the opportunity to use highly sophisticated data-driven approaches at the expense of flexibility and ease of use by non-experts on the one hand, or very simplified models with user-friendly GUIs and no specific computational requirements on the other. Our approach aims at optimizing the balance of complex and sophisticated data-driven epidemic modeling at the global scale while maintaining an accessible computational speed and overall flexibility in the description of the simulation scenario, including the compartmental model, transition rates, intervention measures, and outbreak conditions by means of a user-friendly GUI.

In the GLEaMviz tool the setup of the simulations is highly flexible in that the user can design arbitrary disease compartmental models, thus allowing an extensive range of human-to-human infectious diseases and intervention strategies to be considered. The user interface has been designed in order to easily define both features specific to each compartment, such as the mobility of classes of individuals, and general environmental effects, such as seasonality for diseases like influenza. In addition, the user can define the initial settings that characterize the initial geographical and temporal conditions, the immunity profile of the population, and other parameters including but not limited to: the definition of an outbreak condition in a given country; the number of stochastic runs to be performed; and the total duration of each simulation. The tool allows the production of global spreading scenarios with geographical high resolution by just interacting with the graphic user interface. While an expert input would be required to interpret and discuss the results obtained with the software, the present computational platform facilitates the generation and analysis of scenarios from intensive data-driven simulations. The tool can be deployed both in training activities as well as to facilitate the use of large-scale computational modeling of infectious diseases in the discussion between modelers and public health stakeholders.

The paper is organized as follows. The "Implementation" section describes the software application architecture and its major components, including the computational model GLEaM. The "Results and discussion" section presents in detail the GLEaMviz client and its components that allow for software-user interaction, including an application of the Simulator to an Influenza-like-illness scenario.

## Implementation

**Figure 1:**
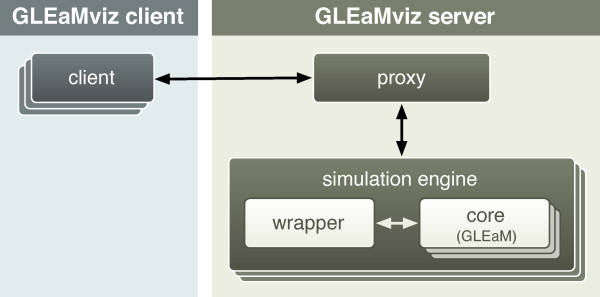
**The GLEaMviz tool system involves one or more GLEaMviz clients that interact with a GLEaMviz server over a TCP connection**. The server consists of a GLEaMviz proxy middleware component and a number of GLEaMviz simulation engines. The middleware component handles the interactions with the clients and manages the engine instances. Each submitted simulation is performed by one GLEaMviz simulation engine instance, which consists of a wrapper that launches the actual simulation cores, one for each run, and performs statistical analysis on the aggregated results.

The top-level architecture of the GLEaMviz tool comprises three components: the GLEaMviz client application, the GLEaMviz proxy middleware, and the simulation engine. The latter two components constitute the GLEaMviz server, as shown in Figure 1.

**Figure 2:**
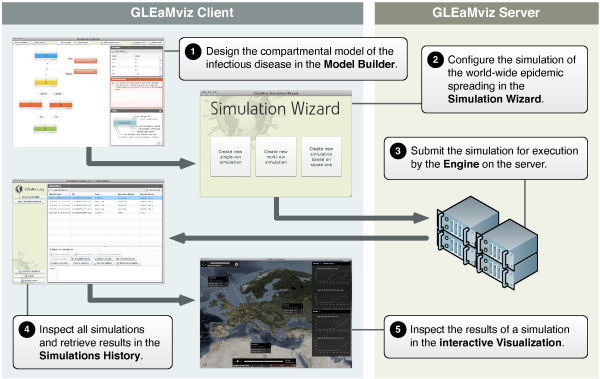
**The principal GLEaMviz tool workflow with the GLEaMviz components involved in each step**. The compartmental model of the infectious disease is created in the Model Builder. Next, the Simulation Wizard is used to configure the simulation and submit it for execution by the Engine on the server. The Simulation History keeps track of the submitted simulations and retrieves the simulation results when they become available. The simulation results are finally inspected in the interactive Visualization.

Users interact with the GLEaMviz system by means of the client application, which provides graphical user-interfaces for designing and managing the simulations, as well as visualizing the results. The clients, however, do not themselves run the simulations. Instead they establish a connection with the GLEaMviz proxy middleware to request the execution of a simulation by the server. Multiple clients can use the same server concurrently. Upon receipt of requests to run a simulation, the middleware starts the simulation engine instances required to execute the requests and monitors their status. Once the simulations are completed, the GLEaMviz proxy middleware collects and manages the resulting simulation data to be served back to the clients. A schematic diagram of the workflow between client and server is shown in Figure 2.

This client-server model allows for full flexibility in its deployment; the client and server can be installed on the same machine, or on different machines connected by a local area network or the Internet. The two-part decomposition of the server in terms of middleware and engines additionally allows for advanced high-volume setups in which the middleware server distributes the engine instances over a number of machines, such as those in a cluster or cloud. This architecture thus ensures high speed in large-scale simulations and does not rely on the CPU-specific availability accessible by the user.

The GLEaMviz simulation engine uses a stochastic metapopulation approach [17,2-22,16] that considers data-driven schemes for the short-range and long-range mobility of individuals at the inter-population level, coupled with coarse-grained techniques to describe the infection dynamics within each subpopulation [6,14]. The basic mechanism for epidemic propagation occurs at multiple scales. Individuals interact within each subpopulation and may contract the disease if an outbreak is taking place in that subpopulation. By travelling while infected, individuals can carry the pathogen to a non-infected region of the world, thus starting a new outbreak and shaping the spatial spread of the disease.

**Figure 3:**
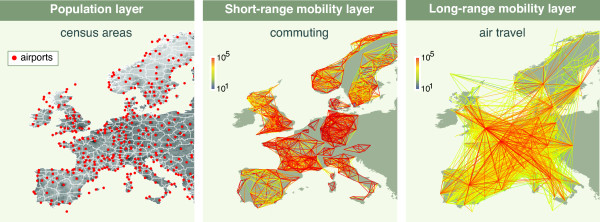
**The three population and mobility data layers in GLEaM**. The *population layer *consists of demographic data at a resolution of 15 × 15 minutes of arc, organized in geo-referenced census areas obtained with a Voronoi tessellation procedure around transportation hubs. The *short-range mobility layer *covers commuting patterns between adjacent subpopulations based on data collected and analyzed from more than 30 countries on 5 continents across the world, modeled with a time-scale separation approach that defines the effective force of infections in connected subpopulations. The *long-range mobility layer *covers the air travel flow, measured in available seats between worldwide airport pairs connected by direct flights.

The basic structure of GLEaM consists of three distinct layers - the population layer, the mobility layer, and the epidemic layer (see Figure 3) [6,14].

The *population layer *is based on the high-resolution population database of the Gridded Population of the World project by the Socio-Economic Data and Applications Center (SEDAC) [23] that estimates population with a granularity given by a lattice of cells covering the whole planet at a resolution of 15 × 15 minutes of arc.

The *mobility layer *integrates short-range and long-range transportation data. Long-range air travel mobility is based on travel flow data obtained from the International Air Transport Association (IATA [24]) and the Official Airline Guide (OAG [25]) databases, which contain the list of worldwide airport pairs connected by direct flights and the number of available seats on any given connection [26]. The combination of the population and mobility layers allows for the subdivision of the world into geo-referenced census areas obtained by a Voronoi tessellation procedure around transportation hubs. These census areas define the subpopulations of the metapopulation modeling structure, identifying 3,362 subpopulations centered on IATA airports in 220 different countries. The model simulates the mobility of individuals between these subpopulations using a stochastic procedure defined by the airline transportation data [6]. Short-range mobility considers commuting patterns between adjacent subpopulations based on data collected and analyzed from more than 30 countries in 5 continents across the world [6]. It is modeled with a time-scale separation approach that defines the effective force of infections in connected subpopulations [6,27,28].

On top of the population and mobility layers lies the *epidemic layer*, which defines the disease and population dynamics. The infection dynamics takes place within each subpopulation and assumes a compartmentalization [29] that the user can define according to the infectious disease under study and the intervention measures being considered. All transitions between compartments are modeled through binomial and multinomial processes to preserve the discrete and stochastic nature of the processes. The user can also specify the initial outbreak conditions that characterize the spreading scenario under study, enabling the seeding of the epidemic in any geographical census area in the world and defining the immunity profile of the population at initiation.

Seasonality effects are still an open problem in the transmission of ILI diseases. In order to include the effect of seasonality on the observed pattern of ILI diseases, we use a standard empirical approach in which seasonality is modeled by a forcing that reduces the basic reproductive number by a factor α_min _ranging from 0.1 to 1 (no seasonality) [20]. The forcing is described by a sinusoidal function of 12 months-period that reaches its peak during Winter time and its minimum during Summer time in each Hemisphere, with the two Hemispheres with opposite phases.

Given the population and mobility data, infection dynamics parameters, and initial conditions, GLEaM performs the simulation of stochastic realizations of the worldwide unfolding of the epidemic. From these *in silico *epidemics a variety of information can be gathered, such as prevalence, morbidity, number of secondary cases, number of imported cases, hospitalized patients, amounts of drugs used, and other quantities for each subpopulation with a time resolution of 1 day.

GLEaM has been under continuous development since 2005 and during these years it has been used: to assess the role of short-range and long-range mobility in epidemic spread [30,31,6]; to retrospectively analyze the SARS outbreak of 2002-2003 in order to investigate the predictive power of the model [22]; to explore global health strategies for controlling an emerging influenza pandemic with pharmaceutical interventions under logistical constraints [21]; and more recently to estimate the seasonal transmission potential of the 2009 H1N1 influenza pandemic during the early phase of the outbreak to provide predictions for the activity peaks in the Northern Hemisphere [3,32].

The GLEaMviz simulation engine consists of a core that executes the simulations and a wrapper that prepares the execution based on the configuration relayed from the client by the GLEaMviz proxy middleware. The engine can perform either single-run or multi-run simulations. The single-run involves only a single stochastic realization for a given configuration setup and a random seed. The multi-run simulation involves a number of stochastic realizations as set by the user and performed by the core (see the following Section), each with the same configuration but with a different random seed. The results of the multi-run simulation are then aggregated and statistically analyzed by the wrapper code.

The simulation engine writes the results to files and uses lock files to signal its status to the middleware component. The core is written in C++, resulting in a fast and efficient engine that allows the execution of a single stochastic simulation of a 1-year epidemic with a standard SEIR model in a couple of minutes on a high-end desktop computer. The wrapper code is written in Python [33]. The server components can be installed on most UNIX-like operating systems such as Linux, BSD, Mac OS X, etc.

The GLEaMviz proxy middleware is the server component that mediates between clients and simulation engines. It accepts TCP connections from clients and handles requests relayed over these connections, providing client authorization management.

A basic access control mechanism is implemented that associates a specific client with the simulations it launches by issuing a private simulation identifier key upon submission. Users can only retrieve the results of the simulations they launched, or simulations for which they have obtained the simulation definition file -containing the private simulation identifier key- from the original submitter.

Upon receipt of a request to execute a simulation, the middleware sets up the proper system environment and then launches an instance of the simulation engine with the appropriate configuration and parameters according to the instructions received from the client. For single-run simulations, the daily results are incrementally served back to the client while the simulation is being executed. This allows for the immediate visualization of the spreading pattern, as described in "Visualization interface" Subsection. For multi-run simulations the results are statistically analyzed after all runs are finished, and the client has to explicitly request the retrieval of the results once they become available. The GLEaMviz proxy server component can be configured to keep the simulation data indefinitely or to schedule the cleanup of old simulations after a certain period of time. Multi-run metadata is stored in an internal object that is serialized on a system file, ensuring that authorization information is safely kept after a server shutdown or failure. The GLEaMviz proxy component additionally provides control features such as accepting administrative requests at runtime in order to manage stored simulations or to modify several configuration parameters like the number of simultaneous connections allowed, the number of simultaneous simulations per client, the session timeout, etc.

The middleware server is written in Python [33] and uses the Twisted Matrix library suite [34] for its networking functionality. Client and server communicate using a special purpose protocol, which provides commands for session handling and simulation management. Commands and data are binary encoded using Adobe Action Message Format (AMF3) in order to minimize bandwidth needs.

The GLEaMviz client is a desktop application by which users interact with the GLEaMviz tool. It provides GUIs for its four main functions: 1) the design of compartmental models that define the infection dynamics; 2) the configuration of the simulation parameters; 3) the visualization of the simulation results; and 4) the management of the user's collection of simulations. In the following Section we describe these components in detail.

The client was developed using the Adobe AIR platform [35] and the Flex framework [36] and can thus be deployed on diverse operating systems, including several Windows versions, Mac OS X, and several common Linux distributions. The GLEaMviz client has a built-in updating mechanism to check for the latest updates and developments and prompts the user to automatically download them. It also offers a menu of configuration options of the interface that allows the user to customize preferences about data storage, visualization options, the server connection, and others.

## Results and Discussion

The software system presented above is operated through the GLEaMviz client, which provides the user interface: the part of the tool actually experienced on the user side. The GLEaMviz client integrates different modules that allow the management of the entire process flow from the definition of the model to the visualization of the results. In the following we will describe the various components and provide the reader with a user study example.

### Model Builder

**Figure 4:**
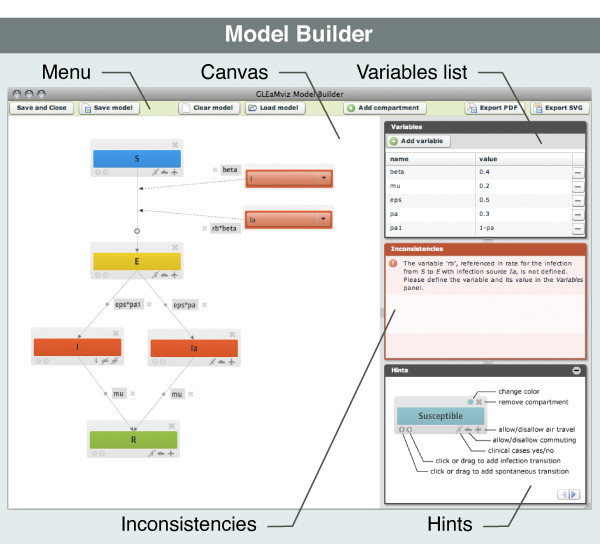
**The compartmental Model Builder allows the user to define the dynamics of the infection by creating population compartments and specifying the infectious and the spontaneous transitions that occur between them**. The Model Builder provides an interactive diagram of the compartmental model, a table with the values for the variables used in the model, a list of current inconsistencies in the model, and a panel that provides concise descriptions on the various functionalities in the interactive diagram.

The Model Builder provides a visual modeling tool for designing arbitrary compartmental models, ranging from simple *SIR *models to complex compartmentalization in which multiple interventions can be considered along with disease-associated complications and other effects. (An example can be found in previous work [37].) A snapshot of the Model Builder window is shown in Figure 4.

**Figure 5:**
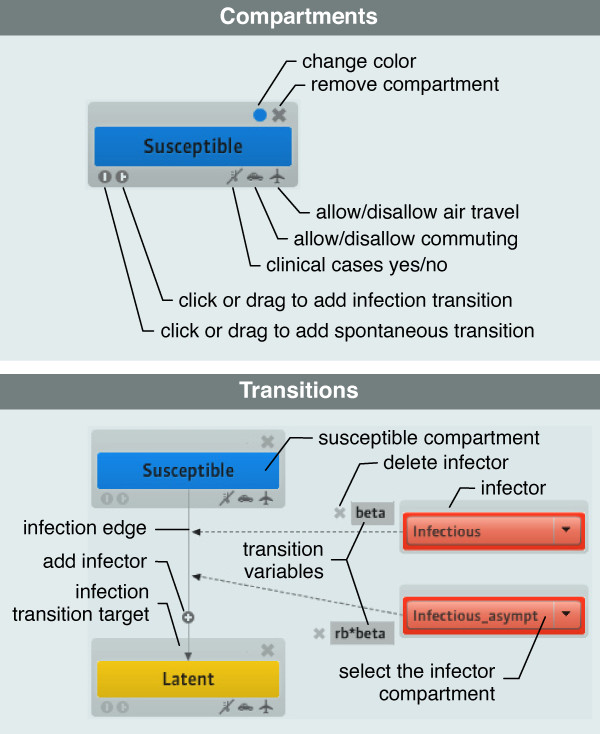
**The interactive compartmental model diagram in the Model Builder represents the compartments and the transitions**. Various user interface elements in these representations allow the user to manipulate the model occurring to his/her needs.

The models are represented as flow diagrams with stylized box shapes that represent compartments and directed edges that represent transitions, which is consistent with standard representations of compartmental models in the literature. Through simple operations like 'click and drag' it is possible to create any structure with full flexibility in the design of the compartmentalization; the user is not restricted to a given set of pre-loaded compartments or transition dynamics. The interactive interface provided by the Model Builder enables the user to define the compartment label, the mobility constraints that apply (e.g. allowed/not allowed to travel by air or by ground), whether the compartment refers to clinical cases, as well as the color and position of their representation in the diagram (see Figure 5). This allows the user to model many kinds of human-to-human infectious diseases, in particular respiratory and influenza-like diseases.

Transitions from one compartment to another can be of two types: infection transitions or spontaneous transitions. Examples of spontaneous transitions include the transitions from latent to infectious individuals and from infectious to recovered individuals. An infection transition, on the other hand, generates new infected individuals. In GLEaM we consider a homogeneous mixing assumption in each subpopulation so that the expected number of new infections generated by *I *infectious individuals in a population with *S *susceptible individuals is equal to $\beta \frac{SI}{N}$, where *N *is the total size of the subpopulation. The GLEaM simulation engine considers discrete individuals. All its transition processes are both stochastic and discrete, and are modeled through binomial and multinomial processes.

Transitions can be visually added by dragging a marker from the source to the target compartment. Spontaneous transitions are annotated with their rates, which can be modified interactively. Infection transitions are accompanied with a representation of the infection's source compartment and the applicable rate (i.e. *β *in the example above), which can also be modified in an interactive way. The rates can be expressed in terms of a constant value or in terms of a variable whose value needs to be specified in the variables table, as shown in Figure 4. The value can also be expressed by simple algebraic expressions.

The client automatically checks for and reports inconsistencies in the model in order to assist the user in the design process (see bottom right window in Figure 4).

Models can be exported to XML files and stored locally, allowing the user to load a model later, modify it, and share it with other users. The diagram representation can be exported as a PDF or SVG file for use in documentation or publications. A few examples of compartmental models are available for download from the Simulator website.

### Simulation Wizard

**Figure 6:**
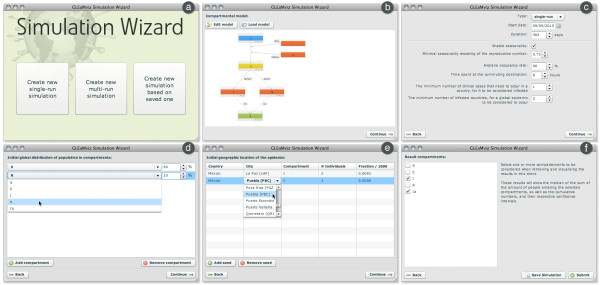
**The Simulation Wizard provides a sequence of panels that leads the user through the definition of the settings and parameters that characterize the simulation**. These panels prompt the user to specify: (a) the type of simulation; (b) the compartmental model; (c) the simulation parameters; (d) the initial distribution of the population amongst the compartments; (e) the initial conditions of the start of the epidemic outbreak; and (f) the compartments considered for the results gathered at the end of the simulation.

The Simulation Wizard provides a sequence of panels that leads the user through the definition of several configuration parameters that characterize the simulation. Figure 6 shows some of these panels. The consecutive steps of the configuration are as follows:

•Choice of the type of the simulation *(panel a)*

The user is prompted with three options: create a new single-run simulation or a new multi-run simulation from scratch, or a new one based on a saved simulation previously stored in a file.

•Compartmental model selection and editing *(panel b)*

The user can design a new compartmental model, modify the current compartmental model (when deriving it from an existing simulation), or load a model compartmentalization from a file.

•Definition of the simulation parameters *(panel c)*

The user is asked to specify various settings and parameter values for the simulation, including, e.g., the number of runs to perform (only accessible in the case of a multi-run), the initial date of the simulation, the length of the simulation (in terms of days), whether or not seasonality effects should be considered, the airplane occupancy rate, the commuting time, the conditions for the definition of an outbreak, and others.

•Initial assignment of the simulation *(panel d)*

Here the user assigns the initial distribution of the population amongst compartments, defining the immunity profile of the global population on the starting date.

•Definition of the outbreak start *(panel e)*

This panel allows the user to define the initial conditions of the epidemic by selecting the city (or cities) seeded with the infection.

•Selection of output results *(panel f)*

Here the user selects the compartments that will constitute the output provided by the client at the end of the simulation. The corresponding data will be shown in the Visualization Window and made available for download.

When all the above configuration settings are defined, the user can submit the simulation to the GLEaMviz server for execution. This will automatically add the simulation to the user's Simulations History. It is furthermore possible to save the definition of the simulation setup to a local file, which can be imported again later or shared with other users.

### Simulations History

**Figure 7:**
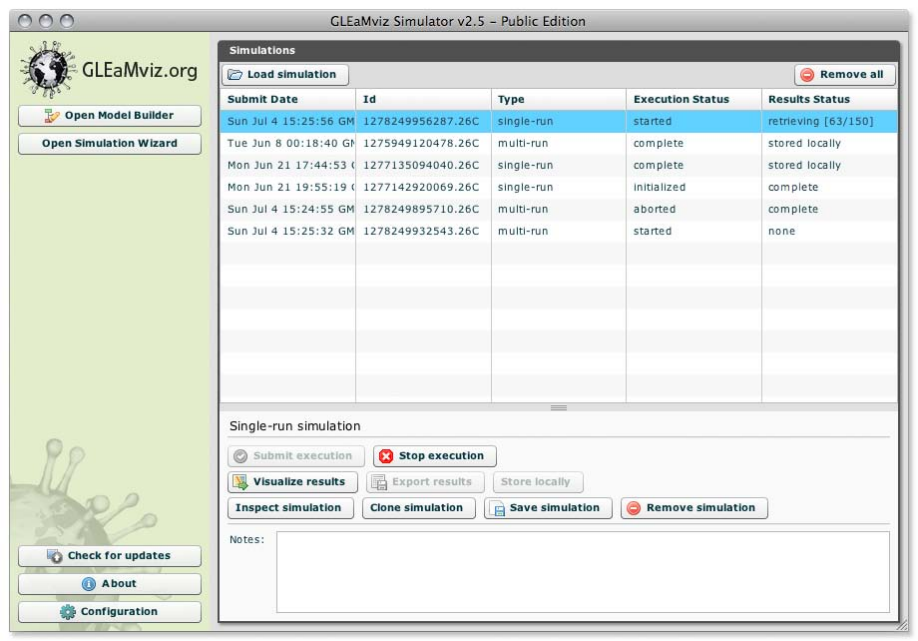
**The main window contains the main menu and the Simulations History, which provides an overview of all the simulations set up by the user, and a contextual menu with the applicable operations**.

The Simulations History is the main window of the client and provides an overview of the simulations that the user has designed and/or submitted, in addition to providing access to the Model Builder, the Simulation Wizard, and the Visualization Component. The overview panel shown in Figure 7 lists the simulation identifier, the submission date and time, the simulation type (i.e., single or multi-run), the execution status (i.e., initialized, start pending, started, aborted, complete, failed, or stop pending) and the results status (i.e., none, retrieve pending, retrieving, stop retrieve pending, complete, or stored locally). Additional File 1 provides a detailed explanation of all these values.

A number of context-dependent command buttons are available once a simulation from the list is selected. Those buttons allow the user to control the simulation execution, retrieve the results from the server and visualize them, clone and edit the simulation to perform a new execution, save the simulation definition or the output data to the local machine (in order to analyze the obtained data with other tools, for example), and remove the simulation. In addition to exporting the compartmental model (see the "Model Builder" Subsection) the user can export a complete configuration of a simulation that includes the compartmental model and the entire simulation setup to a local file, which can be imported again later or shared with other users.

### Visualization interface

**Figure 8:**
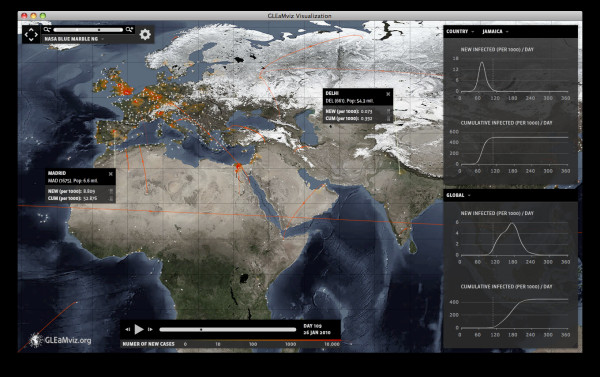
**The simulation results can be inspected in an interactive visualization of the geo-temporal evolution of the epidemic**. The map shows the state of the epidemic on a particular day with infected population cells color-coded according to the number of new cases of the quantity that is being displayed. Pop-ups provide more details upon request for each city basin. The zoomable multi-scale map allows the user to get a global overview, or to focus on a part of the world. The media-player-like interface at the bottom is used to select the day of interest, or show the evolution of the epidemic like a movie. Two sets of charts on the right show the incidence curve and the cumulative size of the epidemics for selectable areas of interest.

Once the execution of a simulation is finished and the results have been retrieved from the server, the client can display the results in the form of an interactive visualization of the geo-temporal evolution of the epidemic. This visualization consists of a temporal and geographic mapping of the results accompanied by a set of graphs (see Figure 8). The geographic mapping involves a zoomable multi-scale map on which the cells of the population layer are colored according to the number of new cases of the quantity that is being displayed. Several visualization features can be customized by clicking on the gear icon and opening the settings widget. It is possible to zoom in and out and pan by means of the interface at the top left of the map. Dragging the map with the mouse (on a location where there are no basin marks) can also pan the visualization. All the widgets and the graphs displayed over the map can be re-positioned according to the user's preferences by clicking and dragging the unused space in the title bar.

The color coding of the map represents the number of cases on a particular day. The time evolution of the epidemic can be shown as a movie, or in the form of daily states by moving forward or backward by one day at a time. For single-run simulations it is also possible to show the airline transportation of the 'seeding' individuals by drawing the traveling edge between the origin and destination cities. In the case where the output quantity is a subset of the infectious compartments, the edges show the actual seeding of the infection. Note that the evolution of the epidemic depends strongly on the model definition. For example, it is possible that some basins are infected by a latent individual that later develops the disease. In this case no seeding flight will be shown if only infectious compartments are selected as output.

Beside the geographical map, the Visualization Window displays two charts. One chart shows the number of new cases per 1,000 over time (incidence), and the other shows the cumulative number of new cases per 1,000 over time (size). For multi-run simulations, median values and corresponding 95% confidence intervals are shown. The menu above each chart combo lets the user choose the context for which the corresponding charts show incidence and size data. This context is either: global, one of three hemispheres, one continent, one region, one country, or one city. The currently selected day is marked by a vertical line in these plots, and the day number, counted from the initial date selected for the simulation, is shown by side of the time slider.

### User study example

Here we present an example application of the GLEaMviz tool to study a realistic scenario for the mitigation of an emerging influenza pandemic. Disease-control programs foresee the use of antiviral drugs for treatment and short-term prophylaxis until a vaccine becomes available [38]. The implementation of these interventions rely both on logistical constraints [21,39] - related, e.g., to the availability of drugs - and on the characteristics of the infection, including the severity of the disease and the virus's potential to develop resistance to the drugs [40].

Here we focus on the mitigation effects of systematic antiviral (AV) treatment in delaying the activity peak and reducing attack rate [41-43,7,8,39,40,3], and assume that all countries have access to AV stockpiles. We consider a scenario based on the 2009 H1N1 influenza pandemic outbreak and feed the Simulator with the set of parameters and initial conditions that have been estimated for that outbreak through a Maximum Likelihood Estimate by using the GLEaM model [3]. The results provided by the present example are not meant to be compared with those contained in the full analysis carried out with GLEaM [3] due to the fact that in the present example we do not consider additional mitigation strategies that were put in place during the early phase of the outbreak, such as the sanitary control measures implemented in Mexico [3,44], or the observed reduction in international travel to/from Mexico [45]. Indeed, the current version of GLEaMviz does not allow for interventions that are geographically and/or temporally dependent. However, these features are currently under development and will be available in the next software release. For this reason the simulation scenario that we study in this application of the Simulator does not aim to realistically reproduce the timing of the spreading pattern of the 2009 H1N1 pandemic. The results reported here ought to be considered as an assessment of the mitigating impact of AV treatment alone, based on the initial conditions estimated for the H1N1 outbreak, and assuming the implementation of the same AV protocol in all countries of the world.

**Figure 9:**
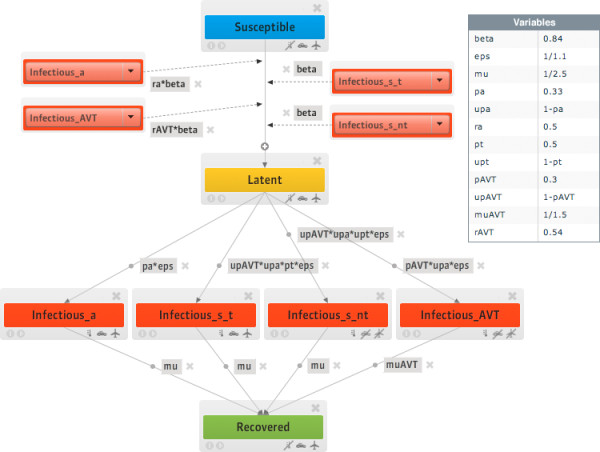
**Compartmental structure in each subpopulation in the intervention scenario**. A modified Susceptible-Latent-Infectious-Recovered model is considered, to take into account asymptomatic infections, traveling behavior while ill, and use of antiviral drugs as a pharmaceutical measure. In particular, infectious individuals are subdivided into: asymptomatic (*Infectious_a*), symptomatic individuals who travel while ill (*Infectious_s_t*), symptomatic individuals who restrict themselves from travel while ill (*Infectious_s_nt*), symptomatic individuals who undergo the antiviral treatment (*Infectious_AVT*). A susceptible individual interacting with an infectious person may contract the illness with rate *beta *and enter the latent compartment where he/she is infected but not yet infectious. The infection rate is rescaled by a factor *ra *in case of asymptomatic infection [41,46], and by a factor *rAVT *in case of a treated infection. At the end of the latency period, of average duration equal to *eps*^-1^, each latent individual becomes infectious, showing symptoms with probability *1-p_a_*, whereas becoming asymptomatic with probability *p_a _*[41,46]. Change in travelling behavior after the onset of symptoms is modeled with probability *p_t _*set to 50% that individuals would stop travelling when ill [41]. Infectious individuals recover permanently after an average infectious period *mu *^-1 ^equal to 2.5 days. We assume the antiviral treatment regimen to be administered to a 30% fraction (i.e. *pAVT *= 0.3) of the symptomatic infectious individuals within one day from the onset of symptoms, reducing the infectiousness and shortening the infectious period of 1 day. [41,42].

We adopt a SEIR-like compartmentalization to model influenza-like illnesses [29] in which we include the systematic successful treatment of 30% of the symptomatic infectious individuals (see Figure 9). The efficacy of the AV treatment is accounted for in the model by a 62% reduction in the transmissibility of the disease by an infected person under AV treatment when AV drugs are administered in a timely fashion [41,42]. We assume that the drugs are administered within 1 day of the onset of symptoms and that the AV treatment reduces the infectious period by 1 day [41,42]. The scenario with AV treatment is compared to the baseline case in which no intervention is considered, i.e. the probability of treatment is set equal to 0 in all countries.

**Figure 10:**
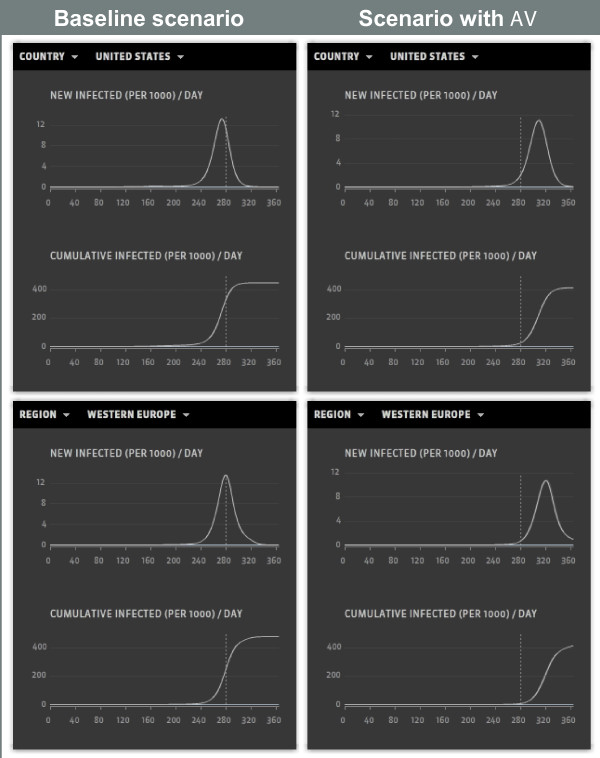
**Simulated incidence profiles for North America and Western Europe in the baseline case (left panels) and in the AV treatment scenario (right panels)**. The plots are extracted from the GLEaMviz tool visualization. In the upper plots of each pair the curves and shaded areas correspond to the median and 95% reference range of 100 stochastic runs, respectively. The lower curves show the cumulative size of the infection. The dashed vertical line marks the same date for each scenario, clearly showing the shift in the epidemic spreading due to the AV treatment.

The GLEaMviz simulation results are shown in Figure 10 where the incidence profiles in two different regions of the world, North America and Western Europe, are shown for both the baseline case and the intervention scenario with AV treatment. The results refer to the median (solid line) and 95% reference range (shaded area) obtained from 100 stochastic realizations of each scenario starting from the same initial conditions. The resulting incidence profiles of the baseline case peak at around mid-November and the end of November 2009 in the US and Western Europe, respectively. These results show an anticipated peak of activity for the Northern Hemisphere with respect to the expected peak time of seasonal influenza. In order to make a more accurate comparison with the surveillance data in these regions, we should rely on the predictions provided by models that can take into account the full spectrum of strategies that were put in place during the 2009 H1N1 outbreak, viz. the predictions obtained by GLEaM [3].

In the case of a rapid and efficient implementation of the AV treatment protocol at the worldwide level, a delay of about 6 weeks would be obtained in the regions under study, a result that could be essential in gaining time to deploy vaccination campaigns targeting high-risk groups and essential services. In addition, the GLEaMviz tool provides simulated results for the number of AV drugs used during the evolution of the outbreak. If we assume treatment delivery and successful administration of the drugs to 30% of the symptomatic cases per day, the number of AV drugs required at the activity peak in Western Europe would be 4.5 courses per 1,000 persons, and the size of the stockpile needed after the first year since the start of the pandemic would be about 18% of the population. Again, we assume a homogeneous treatment protocol for all countries in the world; results may vary from country to country depending on the specific evolution of the pandemic at the national level.

## Conclusions

Computer-based simulations provide an additional instrument for emerging infectious-disease preparedness and control, allowing the exploration of diverse scenarios and the evaluation of the impact and efficacy of various intervention strategies. Here we have presented a computational tool for the simulation of emerging ILI infectious diseases at the global scale based on a data-driven spatial epidemic and mobility model that offers an innovative solution in terms of flexibility, realism, and computational efficiency, and provides access to sophisticated computational models in teaching/training settings and in the use and exploitation of large-scale simulations in public health scenario analysis.

## Availability and requirements

Project name: GLEaMviz Simulator v2.6

Project homepage: http://www.gleamviz.org/simulator/

Operating systems (client application): Windows (XP, Vista, 7), Mac OS X, Linux.

Programming language: C++ (GLEaMsim core), Python (GLEaMproxy, GLEaMsim wrapper), ActionScript (GLEaMviz)

Other requirements (client application): Adobe AIR runtime, at least 200 Mb of free disk space.

License: SaaS

Any restrictions to use by non-academics: None.

The server application can be requested by public institutions and research centers; conditions of use and possible restrictions will be evaluated specifically.

## Competing interests

AV is consulting and has a research agreement with Abbott for the modeling of H1N1 diffusion. The other authors have declared that no competing interests exist.

## Authors' contributions

CG, WVdB and BG designed the software architecture. WVdB and MQ developed the client application. BG implemented the GLEaM engine. CG developed the proxy middleware. CG, VWdB, VC and AV drafted the manuscript. MQ and BG helped draft the manuscript. AV and VC conceived and coordinated the software project, designed and coordinated the application study. All authors read and approved the final manuscript.

## Pre-publication history

The pre-publication history for this paper can be accessed here:

http://www.biomedcentral.com/1471-2334/11/37/prepub

## Supplementary Material

Additional file 1 **The GLEaMviz computational tool: Additional File**. This file includes information for installing the GLEaMviz Client and details of the features of its various components. Click here for file
