# Supplementary material for: The GLEaMviz computational tool, a publicly available software to explore realistic epidemic spreading scenarios at the global scale
Source: BMC Infect Dis. 2011 Feb 2;11:37. doi: 10.1186/1471-2334-11-37 (PMC3048541; doi:10.1186/1471-2334-11-37)
Supplement: Additional file 1 — The GLEaMviz computational tool: Additional File. This file includes information for installing the GLEaMviz Client and details of the features of its various components. [file 1471-2334-11-37-S1.DOC]

**T****he GLEaMviz computational tool, a publicly available software to explore realistic epidemic spreading scenarios at the global scale**

# Additional File

Wouter Van den Broeck^1^, Corrado Gioannini^1^, Bruno Gonçalves^2,3^, Marco Quaggiotto^1,4^, Vittoria Colizza^5,6,7^, Alessandro Vespignani^2,3,7^

^1^ Computational Epidemiology Laboratory, Institute for Scientific Interchange (ISI), Turin, Italy

^2^ Center for Complex Networks and Systems Research, School of Informatics and Computing, Indiana University, Bloomington, IN 47408, USA

^3^ Pervasive Technology Institute, Indiana University, Bloomington, IN 47404, USA

^4^ Department of Industrial Design, Arts, Communication and Fashion (INDACO), Politecnico di Milano, Milan, Italy

^5^ INSERM, U707, Paris F-75012, France

^6^ UPMC Université Paris 06, Faculté de Médecine Pierre et Marie Curie, UMR S 707, Paris F75012, France

^7^ Institute for Scientific Interchange (ISI), Turin, Italy

Here we describe how to install the GLEaMviz client application, how to configure it and how to make use of all its features and functionalities.

1. Client installation

To install the GLEaMviz client two steps are required:

1. download and install the Adobe AIR runtime environment from (if not already installed on the user's local machine):

http://get.adobe.com/air/

2. download and install the GLEaMviz client from:

http://www.gleamviz.org/simulator/

(if the Web browser has the Flash plugin enabled click the "Install now" banner, otherwise click the link to the AIR application file).

Installation is extremely simple and the user is guided by a graphical interface. About 200 MBytes of free disk space are needed to install both the AIR environment and the GLEaMviz client application. Additional disk space is required to store the simulations data locally.

At the installation URL of the GLEaMviz client it is also possible to find a user manual (in PDF) and a video tutorial.

The installation process will add the GLEaMviz tool to the Programs (Windows) or Applications (Mac OSX) list; it will also ask the user if to add a link icon on the Desktop to easily start the program.

2. GLEaMviz client: features details

2.1. Main Window

On the left side of the main window there are the following buttons:

***Check for updates.*** The client has a built-in updating mechanism that periodically connects with the server to check if there are updates available. The user can also request an immediate check for updates by clicking this button.

***About.*** Pressing this button opens a credits screen.

***Configuration.*** This opens the configuration window, which offers the following sections:

1. *Server*, where the user can specify the server parameters; the Public Edition is pre-configured with the proper settings in order to connect to the publicly available server provided by the GLEaMviz project.

2. *System*, where the user can specify some local settings.

3. *Visualization,* where the user can specify options about the Visualization Window.

2.2. Model Builder

The Model Builder window allows the user to easily build a compartmental model with an intuitive interface; it includes the following components:

**Menu. It** contains the buttons that allow the user to edit the model by adding a new compartment, to save or load the model to/from an XML file and to create a local PDF or SVG file displaying the model itself.

**Canvas. It** is made of a WYSIWYG diagram editor that allows the user to build a compartmental model from scratch or modify an existing one: compartments can be added and removed and transition rates between compartments can be defined as simple functions of variables or as constant values.

Compartments are added by clicking on the *Add compartment* button in the menu: a new compartment representation will be added to the canvas with a default name. The name of a compartment can be changed by clicking on the name in that compartment’s representation. Compartment representations can be repositioned by dragging the gray area around the coloured body of the compartment representation. The colour of a compartment can be changed for ease of reference by selecting the colour from the list displayed when the user clicks on the “change colour” button that appears moving the mouse pointer over a compartment representation. On the gray area below the compartment name there are five icons that allow the user to add a transition to another compartment, to mark the compartment as a clinical case and to allow or disallow commuting and air travel for it (see Figure 5 of the main text).

Transitions can be of two types: infection transitions or spontaneous transition. Transitions from one compartment to another can be added by dragging the corresponding icon from the source compartment to the target compartment. Dragging a transition icon to an empty point in the canvas will automatically create a new compartment and a transition of the selected type connecting it. At least one compartment must be defined as corresponding to a “clinical case” by clicking the “thermometer” icon in the compartment interface: this mark is used by the GLEaMviz simulation engine to define the outbreak conditions.

The rate at which a transition process occurs can be set to a variable or to a constant value directly in the canvas, next to each transition arrow.

**Variables.** The variables used in the transition rates in the canvas need to be defined in the variables component on the right side of the Model Builder window: their values can be given as numbers or as simple algebraic expressions involving basic mathematical operators; operands can be either numbers or other variables. It is important to note that only references to previously defined variables are allowed. If a reference to an undefined variable is attempted, the system signals the problem with a message of inconsistency (see below).

**Inconsistencies**. This component highlights the inconsistencies in the model definition found by the client (like e.g. variables or transition rates not defined). Before a model can be saved or used in a simulation definition it must be free of inconsistencies. It is important to note that the client will not check for inconsistencies in the definition of the epidemiological model, which are left to the expertise of the user.

2.3. Simulation Wizard

The Simulation Wizard allows the user to define the parameters characterizing the simulation and to submit it to the GLEaMviz server for execution. This component has been presented in detail in the main text. Here we want to list and describe the simulation parameters that the user can set.

**Simulation parameters.** This interface allows to define the basic scenario for the simulation, in terms of the following parameters:

- *Type*: single-run (SR) or multi-run (MR).
- *Start date*: the date at which the epidemic begins. The default value is the current date.
- *Duration*: the length of each simulation run expressed in days. The default value is 180; the Public Edition imposes a limitation in the duration with a maximum set to 365 days.
- *Number of runs* (MR only): the number of stochastic runs starting from the same initial conditions that must be performed, and over which the statistics of the simulation results will be calculated. The Public Edition imposes a limitation in the number of runs with a maximum set to 10, which is the default value.
- $s_{i}(t)=\frac{1}{2}\left[\left(1-\frac{a_{\min}}{a_{\max}}\right)\sin\left(\frac{2\pi}{365}\left(t-t_{\max,i}\right)+\frac{\pi}{2}\right)+1+\frac{a_{\min}}{a_{\max}}\right]$*Enable seasonality:* if this feature is checked, the GLEaMviz simulation engine will run simulations considering the seasonality effect on the infection transitions. Seasonality is modeled by rescaling the basic reproduction ratio *R*_0_ by a sinusoidal function, *s_i_(t),*

where *i* refers to the hemisphere considered, following the standard approach adopted in the literature [1]. In the tropical region the scaling function is identically equal to 1.0. Along the year the seasonality scaling function varies from a maximum rescaling, $a_{\max}$, to a minimum rescaling $a_{\min}$. $a_{\max}$ is set to 1.1, following previous approaches, whereas $a_{\min}$is set by the user (see below).

- *Minimal seasonality rescaling of the reproductive number* (available only if seasonality is enabled): the minimum value of the rescaling of the reproductive number due to seasonality. The default value is 0.1.
- *Airplane occupancy rate*: the average percentage of seats occupied in each flight. The default value is 90%.
- *Time spent at the commuting destination*: average number of hours spent by commuters at the commuting destination. The default value is 8 hours.
- *Minimum number of clinical cases that need to occur in a country, for it to be considered infected*: this parameter sets the condition for an outbreak in a single country. The default value is set to 1.
- *Minimum number of infected countries, for a global epidemic to be considered to occur*: this parameter sets the condition for the global outbreak. The default value is set to 2.

2.4. Simulations History

The Simulations History is the main panel of the Main Window and provides a list of the simulations that the user has created so far. It reports for each simulation the date and time when the simulation was submitted for execution, its unique identifier, its type and the execution and the results statuses.

**Execution status** can assume the following values:

- *initialized:* the simulation was defined but not yet submitted.
- *start pending:* the execution request has been issued and the client is waiting for a reply.
- *aborted:* the simulation execution has been interrupted by the user.
- *started:* the simulation was successfully submitted and the server is executing it; for single-run simulations the results will be immediately retrieved to be visualized; for multi-run simulations the server will be regularly polled to check the status of the execution of pending simulations.
- *complete:* the simulation execution is complete.
- *failed:* the simulation execution failed.
- *stop pending:* a request to stop the simulation was sent to the server and the client is waiting for a reply.

**Results status** can assume the following values:

- *none:* no results have been retrieved from the server yet.
- *retrieve pending:* a request to retrieve the simulation results was sent to the server and the client is waiting for a reply.
- *retrieving:* the results retrieval is ongoing.
- *stop retrieve pending:* a request to stop the results retrieval has been sent to the server and the client is waiting for a reply.
- *complete:* the results retrieval is complete.
- *stored locally:* the results have been retrieved and stored on the local machine, ready to be displayed without the need to connect to the server.

The following buttons are also available in the **context dependent menu** at the bottom of the Simulations History when selecting a simulation, depending on its status:

- *Submit execution.* Enabled when the execution status is initialized; submits the selected simulation for execution.
- *Stop execution.* Enabled when the execution of the selected simulation is ongoing; sends to the server a request to stop the execution.
- *Check execution status.* Enabled when the execution of the selected multi-run simulation is ongoing; sends to the server a request concerning the execution status of the simulation.
- *Retrieve results.* Enabled when the execution of a multi-run simulation is complete and the results status is none; requests the retrieval of the simulation's results.
- *Visualize results.* Enabled when the execution is complete and the results have been completely retrieved (and/or stored locally); opens the Visualization Window.
- *Export results.* Enabled when the execution is complete and the results have been completely retrieved; exports all results, including relevant metadata and the simulation definition, to a local folder.
- *Store locally/Do not store locally.* Enabled when the simulation's results have been retrieved; selects if to save the result data on the local machine.
- *Inspect simulation.* Opens a read-only version of the Simulation Wizard that shows the model and all the simulation’s parameters.
- *Clone simulation.* Opens the Simulation Wizard with pre-compiled parameters based on the selected simulation.
- *Save simulation.* Exports the simulation definition to an XML file; this file can later be used as the basis for a new simulation or shared with other users.
- *Remove simulation.* Deletes the selected simulation.

3. Exported results

By clicking the *Export results* button in the context dependant menu below the Simulations History, the user can save locally the output results of the simulation. The client will ask the user to select a local directory, under which a new directory named data_<Id> will be created, where <Id> is the Id of the simulation. This directory will contain the following files and subdirectories:

- simulation.xml: the simulation definition (including the model specification, the parameters settings and the initial conditions); this file can be used as an import file in the Simulation Wizard;
- md_cities.tsv: the metadata for the cities-level aggregated output, mapping the numerical identifiers used for the actual output files;
- md_countries.tsv: the metadata for the countries-level aggregated output, mapping the numerical identifiers used for the actual output files;
- md_regions.tsv: the metadata for the regions-level aggregated output, mapping the numerical identifiers used for the actual output files;
- md_continents.tsv: the metadata for the continents-level aggregated output, mapping the numerical identifiers used for the actual output files;
- md_hemispheres.tsv: the metadata for the hemispheres-level aggregated output, mapping the numerical identifiers used for the actual output files;
- cities: a directory containing one .tsv output file for each city, named with the city_id as defined in the metadata file;
- countries: a directory containing one .tsv output file for each country, named with the country id as defined in the metadata file;
- regions: a directory containing one .tsv output file for each region, named with the region id as defined in the metadata file;
- continents: a directory containing one .tsv output file for each continent, named with the continent id as defined in the metadata file;
- hemispheres: a directory containing one .tsv output file for each hemisphere, named with the hemisphere id as defined in the metadata file;
- global.tsv: the output file for the whole globe.

All the .tsv files are Tab Separated Values (TSV). The output TSV files contain three columns for single-run simulations and seven columns for multi-run simulations. The data columns, listed in order, represent the following quantities:

- the time step, corresponding to the day of the simulation starting from 0;
- the new number of individuals per 1000;
- (multi-run only) the lower bound of the corresponding 95% reference range of the stochastic realization;
- (multi-run only) the upper bound of the corresponding 95% reference range of the stochastic realization;
- the cumulative number of individuals per 1000;
- (multi-run only) the lower bound of the corresponding 95% reference range of the stochastic realization;
- (multi-run only) the upper bound of the corresponding 95% reference range of the stochastic realization.

**References**

[1] Cooper BS, Pitman RJ, Edmunds WJ, Gay N: **Delaying the international spread of pandemic influenza.** *PloS Medicine* 2006 , **3:**e12.
